# Evaluation and validation of 2D biomechanical models of the knee for radiograph-based preoperative planning in total knee arthroplasty

**DOI:** 10.1371/journal.pone.0227272

**Published:** 2020-01-08

**Authors:** Malte Asseln, Jörg Eschweiler, Adam Trepczynski, Philipp Damm, Klaus Radermacher

**Affiliations:** 1 Chair of Medical Engineering, Helmholtz-Institute for Biomedical Engineering, RWTH Aachen University, Aachen, Germany; 2 Department of Orthopaedics, Aachen University Clinic, RWTH Aachen University, Aachen, Germany; 3 Julius Wolff Institute, Charité – Universitätsmedizin Berlin, corporate member of Freie Universität Berlin, Humboldt-Universität zu Berlin, and Berlin Institute of Health, Berlin, Germany; Toronto Rehabilitation Institute - UHN, CANADA

## Abstract

Thorough preoperative planning in total knee arthroplasty is essential to reduce implant failure by proper implant sizing and alignment. The “gold standard” in conventional preoperative planning is based on anterior-posterior long-leg radiographs. However, the coronal component alignment is still an open discussion in literature, since studies have reported contradictory outcomes on survivorship, indicating that optimal individual alignment goals still need to be defined. Two-dimensional biomechanical models of the knee have the potential to predict joint forces and, therefore, objectify therapy planning. Previously published two-dimensional biomechanical models were evaluated and validated for the first time in this study by comparison of model predictions to corresponding *in vivo* measurements obtained from telemetric implants for a one- and two-leg stance. Model input parameters were acquired from weight-bearing anterior-posterior long-leg radiographs and statistical assumptions for patient-specific model adaptation. The overall time from initialization to load prediction was in the range of 7–8 minutes per patient for all models. However, no model could accurately predict the correct trend of knee joint forces over patients. Two dimensional biomechanical models of the knee have the potential to improve preoperative planning in total knee arthroplasty by providing additional individual biomechanical information to the surgeon. Although integration into the clinical workflow might be performed with acceptable costs, the models’ accuracy is insufficient for the moment. Future work is needed for model optimization and more sophisticated modelling approaches.

## Introduction

Preoperative planning in total knee arthroplasty (TKA) is increasingly relevant to reduce intraoperative errors related to implant sizing and bony resections, and provides the opportunity to prepare the surgical instrumentation and access to the implant system [1]. The global “gold standard” in clinical preoperative planning in TKA is based on the two-dimensional (2D) geometrical analysis of anterior-posterior (AP) standing long-leg radiographs [2]. References, such as the anatomical and mechanical axes, are measured and implant templates are aligned in a neutral or anatomical mechanical alignment. The templating has been traditionally performed on radiographic films, whereas computer-assisted orthopaedic templating software packages are increasingly available using computed and digital radiography. However, optimal individual alignment goals are still being discussed controversially [3]. Thereby, mechanical factors play an important role in the clinical success of the prosthesis, effecting, for example, wear, loosening and instability and, consequently, implant durability and patient satisfaction [4–6].

The mechanical alignment tries to establish mechanical equilibrium of the medial and lateral compartment in the coronal plane, minimizing shear forces at the prosthetic surface and, thus, theoretically maximizing longevity. However, the medial load share in the normal and prosthetic knee is usually greater than the lateral [7–9]. An equal loading in an asymmetric tibial component might even be unintended. The analysis of 6,070 TKAs showed optimal survivorship for a total of anatomical knee alignment between 2.4° to 7.5° of valgus [10]. Kim et al. [11] reviewed 3,048 knees and defined a similar target zone of 3° to 7.5° of valgus alignment. This is supported by other studies that correlated increased revision rates with coronal malalignment, particularly in varus [12,13]. Bellemans et al. [14] questioned whether neutral mechanical alignment in TKA is normal for all patients after discovering that natural alignment is at least 3° varus at the end of growth for a quarter of the population. Additionally, recent studies have shown that indoctrinated neutral mechanical alignment is probably insufficient [15,16]. Nowadays, patients with severe varus deformity are often left in slight varus and valgus patients *vice versa*. However, the surgeon usually has no information on acting joint forces during preoperative planning, thus, the procedure depends greatly on the expert’s experience.

Validated computer-based modelling approaches have the potential to predict joint forces and, thus, optimize preoperative planning [17]. Three-dimensional numerical models can be classified into two main categories: finite element (FE) and multibody simulation models. The FE models are commonly used to predict joint contact stresses. However, this approach is rather unsuitable for clinical application because of the input data required, such as external load and motion profiles [18], segmented bony surfaces and soft tissues, and their intensive computational costs, which can be in the range of a few days [19]. Multibody simulation models are computationally efficient, but typically idealized joint kinematics are used [20] and the patient-specific adaptation process, for example, based on gait lab data, is also very expensive [21]. Alternatively, 2D analytical models are efficient and robust, and patient-specific adaptation can be performed based on radiographs. A lot of such models have been described in the literature during the last few decades. However, their broad application in preoperative TKA planning is missing. The major reasons are their simplification and lack of validation [22].

The goal of this study was to evaluate the suitability of 2D biomechanical models for radiograph-based preoperative planning in TKA and validate their predictions based on *in vivo* measurements of nine patients treated with instrumented knee implants.

## Materials and methods

### 2D biomechanical models

**Fig 1:**
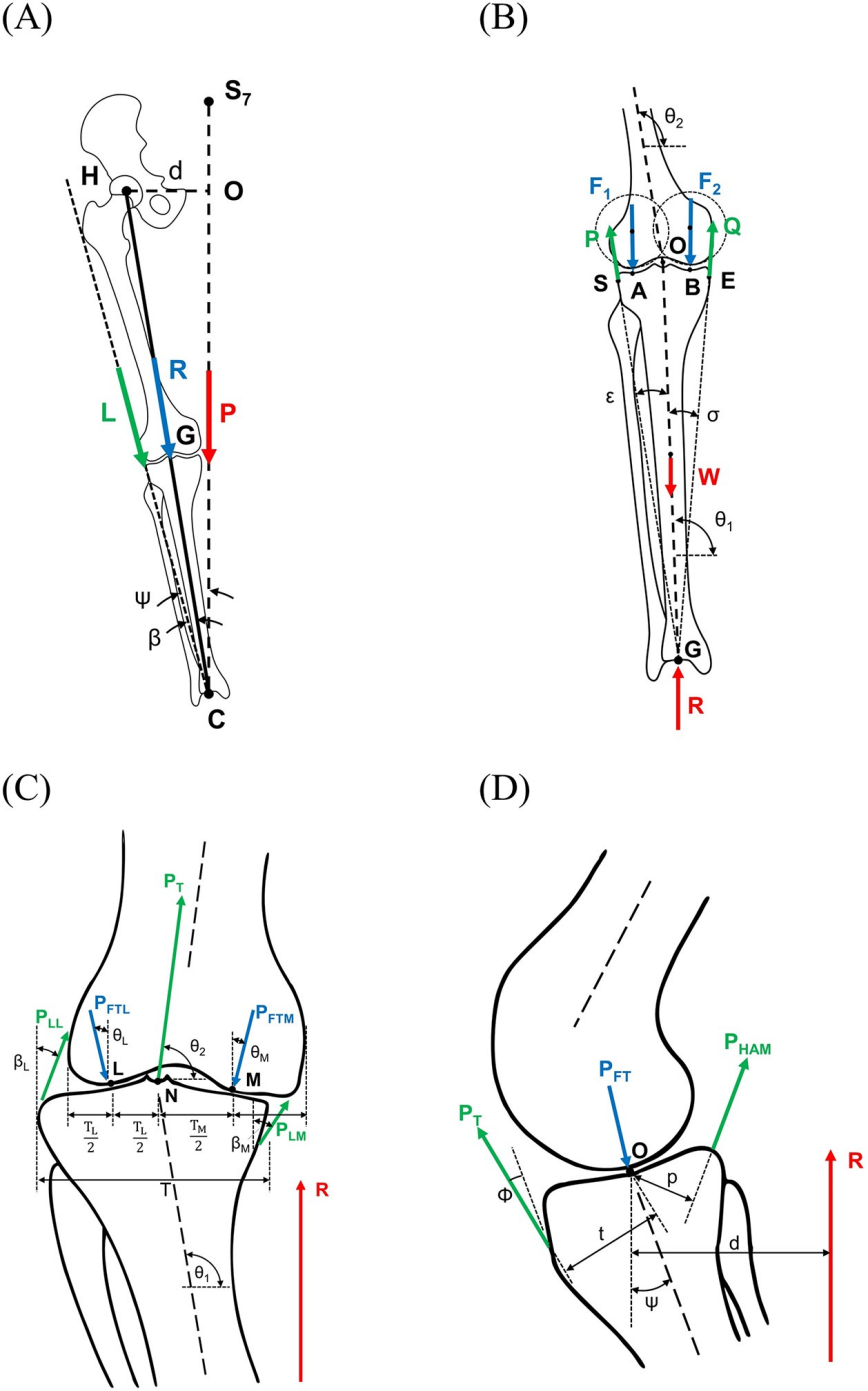
(A) Maquet’s model. (B) Kettelkamp’s model. Minns’ model (modified) [25] in frontal view (C) and sagittal view (D). Joint forces (blue), external and body forces (red), soft-tissue and muscular forces (green). Abbreviations of the forces: (A) L = lateral muscular force; R = knee joint force; P = partial body weight; (B) P = lateral ligament force; Q = medial ligament force; F_1_ = lateral knee joint force; F_2_ = medial knee joint force; W = gravity force of the leg; R = ground reaction force; (C) P_LL_ = lateral ligament force; P_LM_ = medial ligament force; P_FTL_ = lateral knee joint force; P_FTM_ = medial knee joint force; P_T_ = patellar ligament force; R = ground reaction force; (D) P_T_ = patellar ligament force; P_FT_ = knee joint force; P_HAM_ = hamstring force; R = ground reaction force.

An extensive literature research on 2D biomechanical models was performed following Hefzy and Grood [22]. The models of Maquet [23], Kettelkamp [24], and Minns [25] showed a high potential for application based on the requirements to use data available in the conventional clinical workflow for patient-specific model adaptation and allow an easy integration into preoperative planning. All of them focus on the tibiofemoral joint (TF) (Fig 1). Maquet’s model uses one-leg stance AP long-leg radiographs, including pelvis, as input and predicts the resultant knee joint force, its orientation and a lateral muscle force. Kettelkamp’s model was intended to calculate the force distribution on the tibial plateau in the context of proximal tibial osteotomy. However, Kettelkamp stated that it is not only restricted to this application. It simulates a two-leg stance based on two-leg stance AP long-leg radiographs and the outputs are medial and lateral knee joint forces and their orientation. Minns developed an analytical model to investigate the effect of anatomical variations on the medial and lateral knee contact forces. Similar to Maquet, the adaptation process relies on AP long-leg radiographs in a one-leg stance, but it also considers the patient’s anatomy in the sagittal plane. A one-leg stance AP long-leg radiograph and a sagittal radiograph are required for Minn’s model. The medial and lateral knee joint forces are then calculated.

The model provisions were transferred, where possible, to a one-leg stance and a two-leg stance *vice versa* to make the predictions comparable. Additionally, the models vary slightly in terms of the in- and output data required. Weight-bearing two-leg long-leg radiographs, covering the areas of the hip and ankle joint centres, were assumed as standardized input to acquire model-specific geometrical parameters for patient-specific adaptation. Therefore, minor model adaptations were partly inevitable. The modifications and assumptions made in this study are summarized in Table 1. All models have been implemented in MATLAB (The MathWorks, Inc., USA).

**Table 1:** Assumptions made for modelling.

Parameter	Maquet	Kettelkamp	Minns
**Partial body weight**	*P* = 0.929 ∙ *BW* (two-leg) [26] and *P* = 0.429 ∙ *BW* (one-leg) [26]	Tibia *W* = 0.07 ∙ BW [26]	Tibia *W* = 0.06 ∙ BW [25]
**Ground reaction force**		*R* = *BW* (two-leg) and *R* = 0.5 ∙ *BW* (one-leg)	*R* = *BW*(one-leg) and *R* = 0.5 ∙ *BW* (two-leg)
**Force magnitudes**			Patella ligament tension *PT* ≈ *BW* [25]
**Force orientations**			*βL*, *βM* = 0° [25] Ɵ*L*, Ɵ*M* = 0° [25]
**Angles**			*ϕ* = 11° [27] *ψ* = 20° [25]
**Distances**	$d=\frac{0.191}{2}∙BH$ [28]		

### Experimental data

Published data from the public OrthoLoad database (www.orthoload.com) containing loads acting in orthopaedic implants have been used to evaluate and validate the models presented. No additional measurements were performed for this study. All clinical studies were approved by the ethics committee of Charité Universitätsmedizin Berlin. Postoperative weight bearing AP long-leg radiographs were available for a total of nine patients treated with instrumented knee implants for *in vivo* joint force measurements. Patient information and anthropometric data are listed in Table 2. Predominantly elderly (mean age 67.89 years) patients participated and the leg alignment was in slight varus (7 of 9). The telemetric implants are based on the INNEX knee system (Zimmer GmbH, Winterthur, Schweiz), which is designed with an ultra-congruent tibia inlay, sacrificing the cruciate ligaments [29].

**Table 2:** Anthropometric data of the TKA patients.

Patient	Gender	Body weight [kg]	Body size [cm]	Age at implantation [years]	Tibiofemoral angle [°]
**K1L**	Male	105	177	63	3.0 (Varus)
**K2L**	Male	92	171	71	5.0 (Varus)
**K3R**	Male	97.9	175	70	3.5 (Varus)
**K4R**	Female	100.6	170	63	4.5 (Valgus)
**K5R**	Male	96	175	60	1.0 (Varus)
**K6L**	Female	83	174	65	4.0 (Valgus)
**K7L**	Female	69.1	166	74	6.5 (Varus)
**K8L**	Male	79	174	70	4.0 (Varus)
**K9L**	Male	109.1	166	75	7.0 (Varus)

### Patient-specific model adaptation

**Fig 2:**
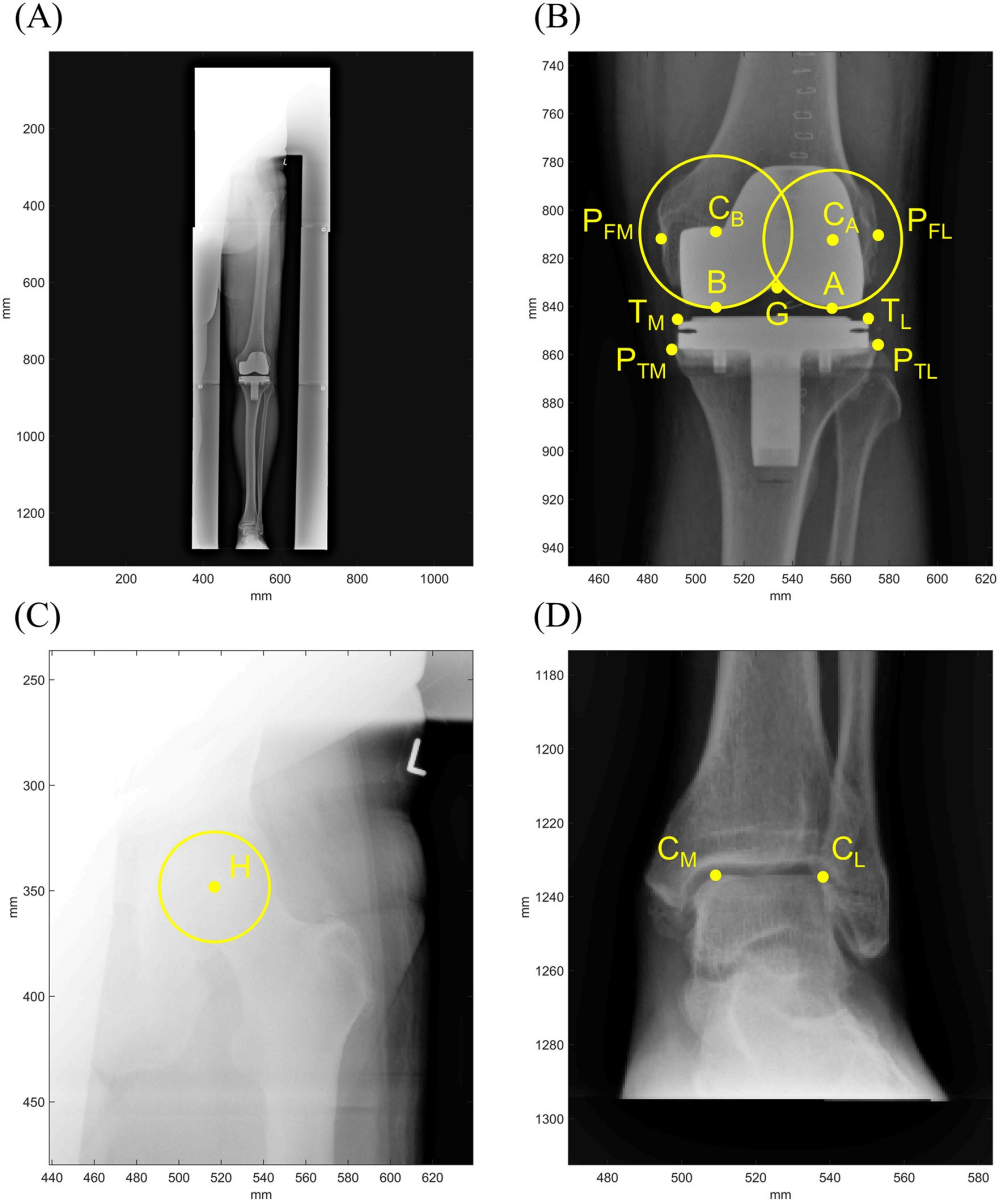
Parameter acquisition for patient-specific model adaptation (exemplary). (A) Long-leg radiograph, (B) knee region, (C) hip region, and (D) ankle region. The nomenclature is presented in Table 3.

**Fig 3:**
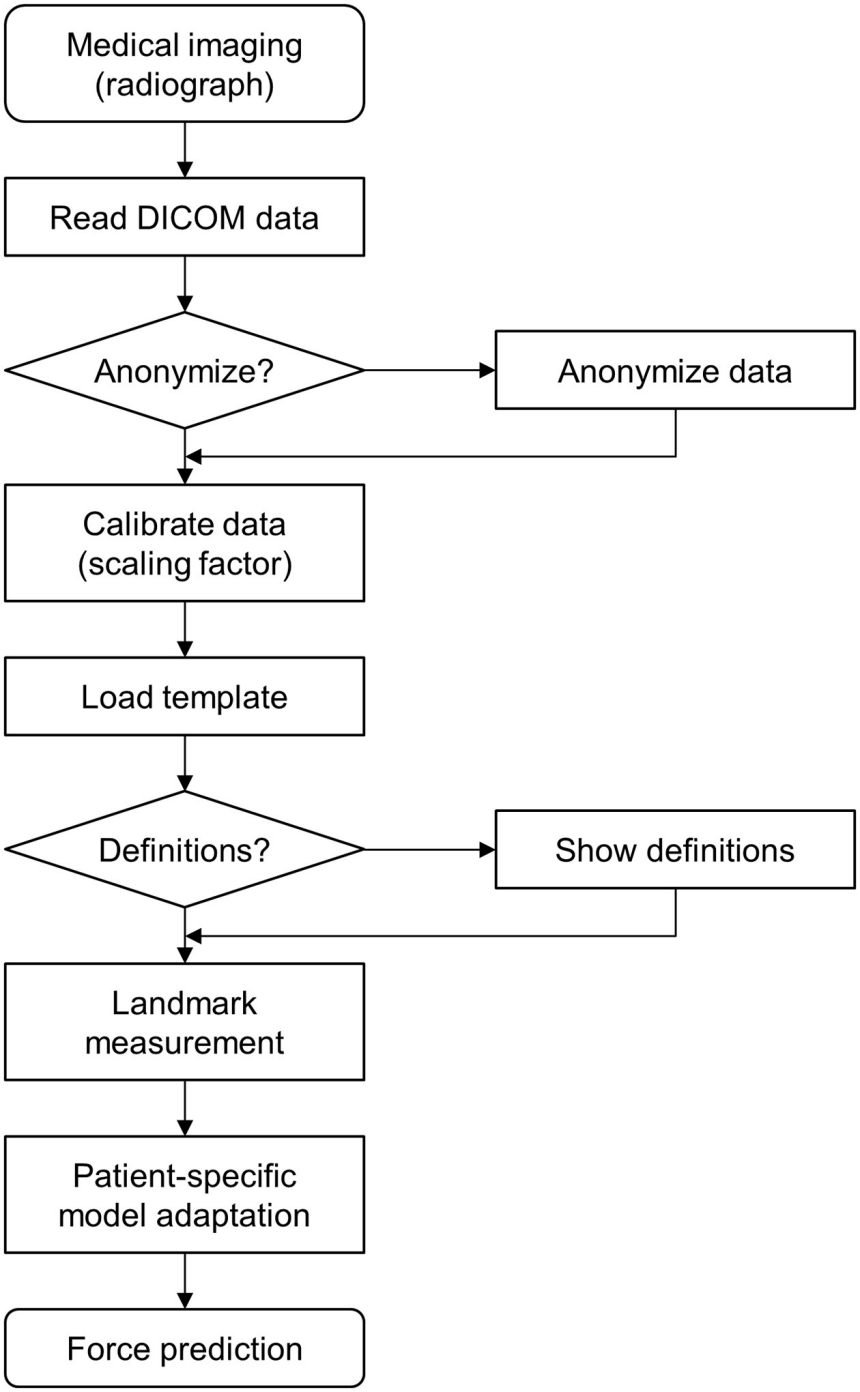
Rule-based protocol for the modelling process. At least three test cycles were performed to reduce learning curve effects.

Model-specific geometrical parameters for the patient-specific model adaptation were acquired from the appropriate postoperative radiographs. Therefore, landmarks were manually measured in the radiographs by a single trained observer based on a self-developed program in MATLAB (Fig 2). A rule-based protocol was used to ensure a systematic and reproducible data acquisition (Fig 3). Each landmark was visualized in an example dataset with corresponding definitions. The template consisted of a total of 15 landmarks for all models, covering a one- and two-leg stance (Table 3). Several test trials were performed by the observer before data acquisition to minimize learning curve effects. Model input parameters were calculated after the measurements.

**Table 3:** Landmark template for the calculation of model-specific input parameters. The circle tool was used for H, C_A_ and C_B_ and the point tool for all other landmarks.

Landmark	Definition	Maquet	Kettelkamp	Minns
C_L_	Lateral point talus plateau	X	X	X
C_M_	Medial point talus plateau	X	X	X
G	Centre knee joint	X	X	X
H	Centre hip joint	X	X	X
F	Midpoint of distal femoral shaft			X
C_A_	Centre of curvature lateral femoral condyle		X	
C_B_	Centre of curvature medial femoral condyle		X	
A	Lateral contact point		X	
B	Medial contact point		X	
P_TM_	Most medial point tibial plateau		X	X
P_TL_	Most lateral point tibial plateau		X	X
P_FL_	Most lateral point femur condyle			X
P_FM_	Most medial point femur condyle			X
T_L_	Most proximolateral point tibial plateau	X	X	X
T_M_	Most proximomedial point tibial plateau	X	X	X

The MATLAB program can import, calibrate and anonymize DICOM images and different tools, such as measure points, distances, circles and angles, are available. Three points were clicked around the femoral head and a circle was fitted to determine the hip joint centre (Fig 2C). The same tool was used to identify the centres of curvature of the two articular surfaces for Kettelkamp’s model to obtain the lines of action of the medial and lateral contact forces (Fig 2B). The ankle joint centre was measured as the mid-width of the talus (Fig 2D). Since some model input parameters correspond to each other, they have been determined only once.

The time was recorded for every measurement and the mean was calculated to assess additional expenses potentially required due to the incorporation of mathematical models of the knee in the TKA preoperative planning process. This was done after the following steps: initialization of the program (import and calibration of radiograph, load of landmark template, etc.), identification of landmarks and final model application (including figure export for documentation).

### Model validation

The three models were adapted to the patient-specific situations and the computational results were compared to the corresponding *in vivo* measurements of a one- and two-leg stance for validation. Thereby, representative reference values were obtained separately.

**Fig 4:**
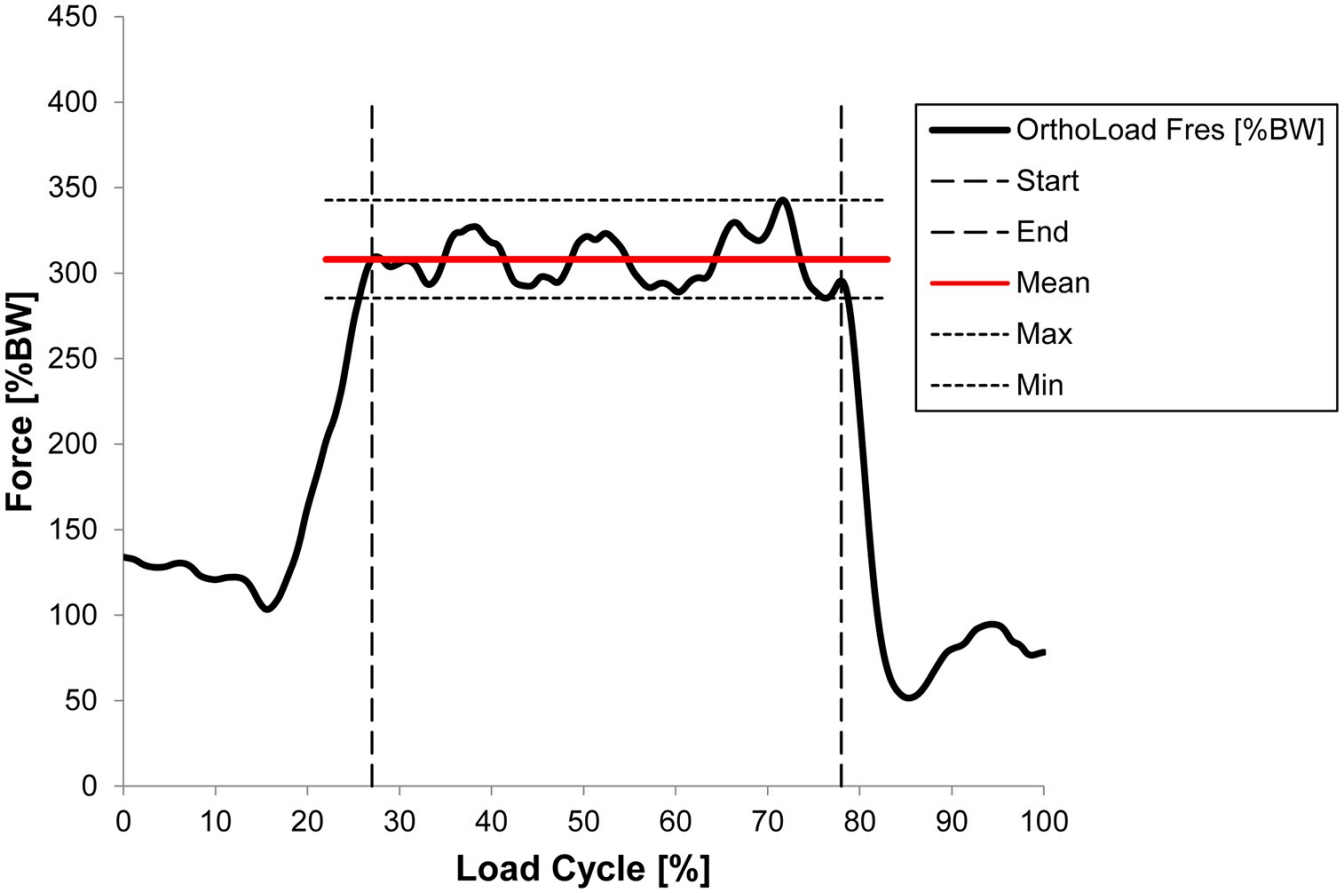
Exemplarily calculation of the average *in vivo* force in a one-leg stance.

Regarding a one-leg stance, the data published in the OrthoLoad database under “Standard Loads Knee Joint” have been used [29]. These contain averages of several trials of eight subjects, excluding K4R. Reference values were calculated by averaging the resultant force data F_res_ over the load cycle with clearly static conditions for each subject (Fig 4). The two-leg stance data were collected from representative two-leg stance where corresponding ground reaction forces of a single leg showed a maximum deviation of 5% from half of the body weight. Here, averages of the resultant knee joint force F_res_ were calculated from three trials.

All joint forces were stated as a percent of the patient’s body weight (%BW) to allow a standardized comparison. The root-mean-square errors (RMSEs) were calculated to measure the accuracies between the three model predictions and the average experimental observation for each subject. In the following, the errors are listed in %BW and, additionally, the absolute difference in percent of the average *in vivo* forces.

Furthermore, correlation and regression analysis were performed between implant alignment, in terms of varus/valgus angles, and medial force ratio, defined as the percentage of the knee joint force that is transferred via the medial compartment.

## Results

All radiographs could easily be imported into the MATLAB program developed. The time for initialization was in the range of 2 min, landmark measurement in the range of 3 to 4 min and model application in the range of 2 min, resulting in a total duration of 7 to 8 min for the entire process. These values already consider minor corrections, for example, in the identification of landmarks.

### Two-leg stance

**Fig 5:**
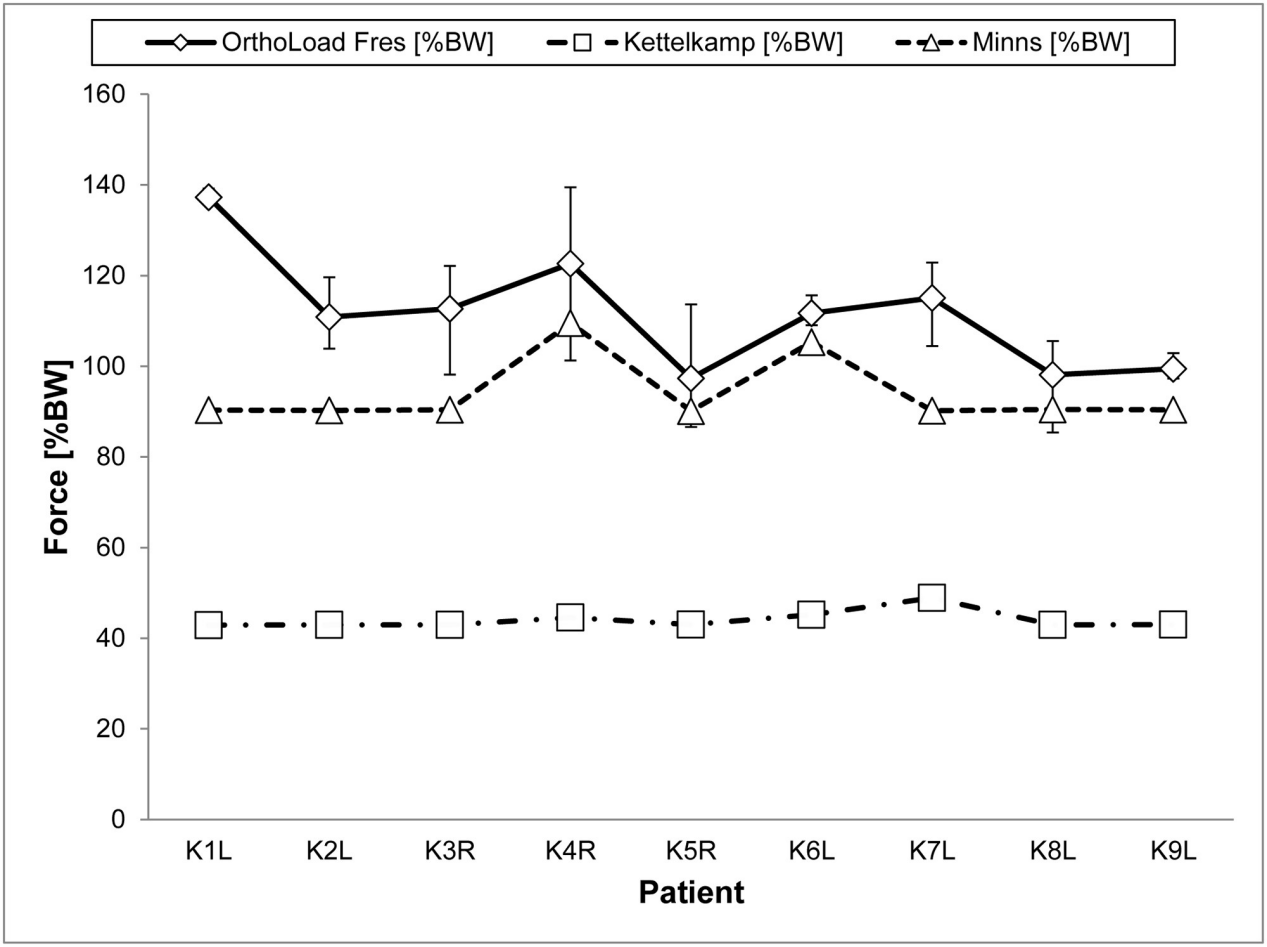
Resultant knee joint forces calculated for nine patients, based on the mathematical models of Kettelkamp and Minns with corresponding average *in vivo* force measurements for a two-leg stance. The error indicators demonstrate the minimum/maximum values. Forces in %BW.

The comparison of the simulated and measured knee joint forces for a two-leg stance are presented in Fig 5. Minns’ model shows values of similar magnitudes, whereas Kettelkamp’s model underestimates by around half of the actuals *in vivo* measurements. Looking at the reference measurements, K1L particularly stands out due to its high value of 137.26%BW. As mentioned in the model description in S1 Appendix, Maquet’s model was excluded because of the fixed model definitions for a one-leg stance. Looking at the numerical values (Table 4), the RMSE was 21.47%BW for Minns’ and 68.70%BW for Kettelkamp’s model, underlining the graphical representation. The percentage deviations revealed similar results with 14.99% and 60.17%, respectively. The force distribution for the medial and lateral side is also noticeable. Patients K4R and K6L in Minns’ model showed a lift off on the medial side, indicated by a medial contact force of zero. There was a lift off in Kettelkamp’s model of patients K4R and K7L on the medial and lateral side, respectively.

**Table 4:** Calculated and observed *in vivo* values during two-leg stance, split into medial, lateral and resultant contact forces F1, F2 and FR. Differences between FR and the average reference values are expressed in terms of RMSE. Forces in %BW, RMSE in %BW and mean differences in % of the average *in vivo* forces.

Patient		K1L	K2L	K3R	K4R	K5R	K6L	K7L	K8L	K9L	
**OrthoLoad Fres [%BW]**		137.26	110.84	112.67	122.65	97.35	111.72	115.07	98.12	99.41	
**Kettelkamp**	**F1 (lateral) [%BW]**	11.60	15.96	20.41	44.52	11.50	41.58	0.00	28.27	7.67	
	**F2 (medial) [%BW]**	31.30	26.96	22.50	0.00	31.51	3.60	48.89	14.66	35.33	
	**FR [%BW]**	42.90	42.92	42.91	44.52	43.01	45.18	48.89	42.93	43.00	
	**Residuals [%BW]**	94.36	67.92	69.76	78.13	54.34	66.54	66.18	55.19	56.41	
	**RMSE [%BW]**										68.70
	**Mean [%]**										60.17
**Minns**	**F1 (lateral) [%BW]**	37.31	41.17	46.64	109.51	35.26	105.35	18.58	51.00	33.55	
	**F2 (medial) [%BW]**	52.99	49.08	43.75	0.00	54.89	0.00	71.63	39.43	56.86	
	**FR [%BW]**	90.30	90.25	90.37	109.51	90.14	105.35	90.21	90.43	90.40	
	**Residuals [%BW]**	46.96	20.59	22.30	13.14	7.21	6.37	24.86	7.69	9.01	
	**RMSE [%BW]**										21.47
	**Mean [%]**										14.99

The resultant knee joint forces for both models were mainly of the same order of magnitude for all subjects of 44%BW and 90%BW, respectively. K4R and K6L were exceptions only in Minn’s model, with knee joint forces higher than 100%BW. However, large variations were observed looking at the medial/lateral load distribution. Furthermore, similar model output in %BW means different calculations in the absolute force due to patient-specific differences in body weight.

**Fig 6:**
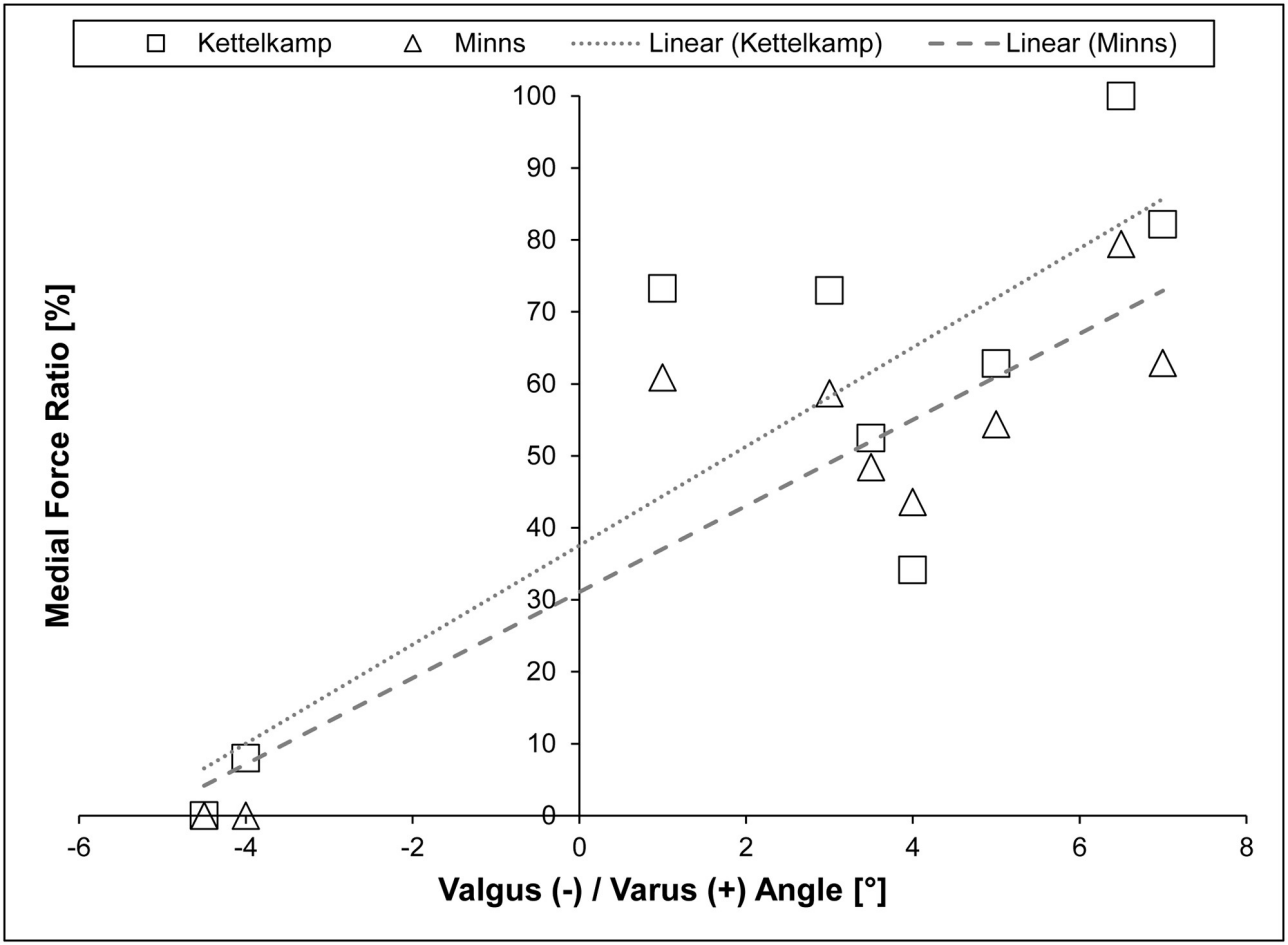
Correlation and regression analysis between tibiofemoral angle and medial force ratio during static two-leg stance based on the models of Kettelkamp and Minns.

The correlations between the implant alignment and the predicted compartment forces for a two-leg stance are shown in Fig 6. It can be seen that the medial force ratio was higher for larger tibiofemoral angles, and *vice versa*. The Pearson’s correlation coefficient was 0.85 for Kettelkamp’s and 0.90 in Minns’ model, indicating a high correlation. Looking at the linear regression lines, an equilibrium of the predicted medial and lateral contact forces was established at varus angles of 1.8° for Kettelkamp’s model and 3.2° for Minns’, respectively.

### One-leg stance

**Fig 7:**
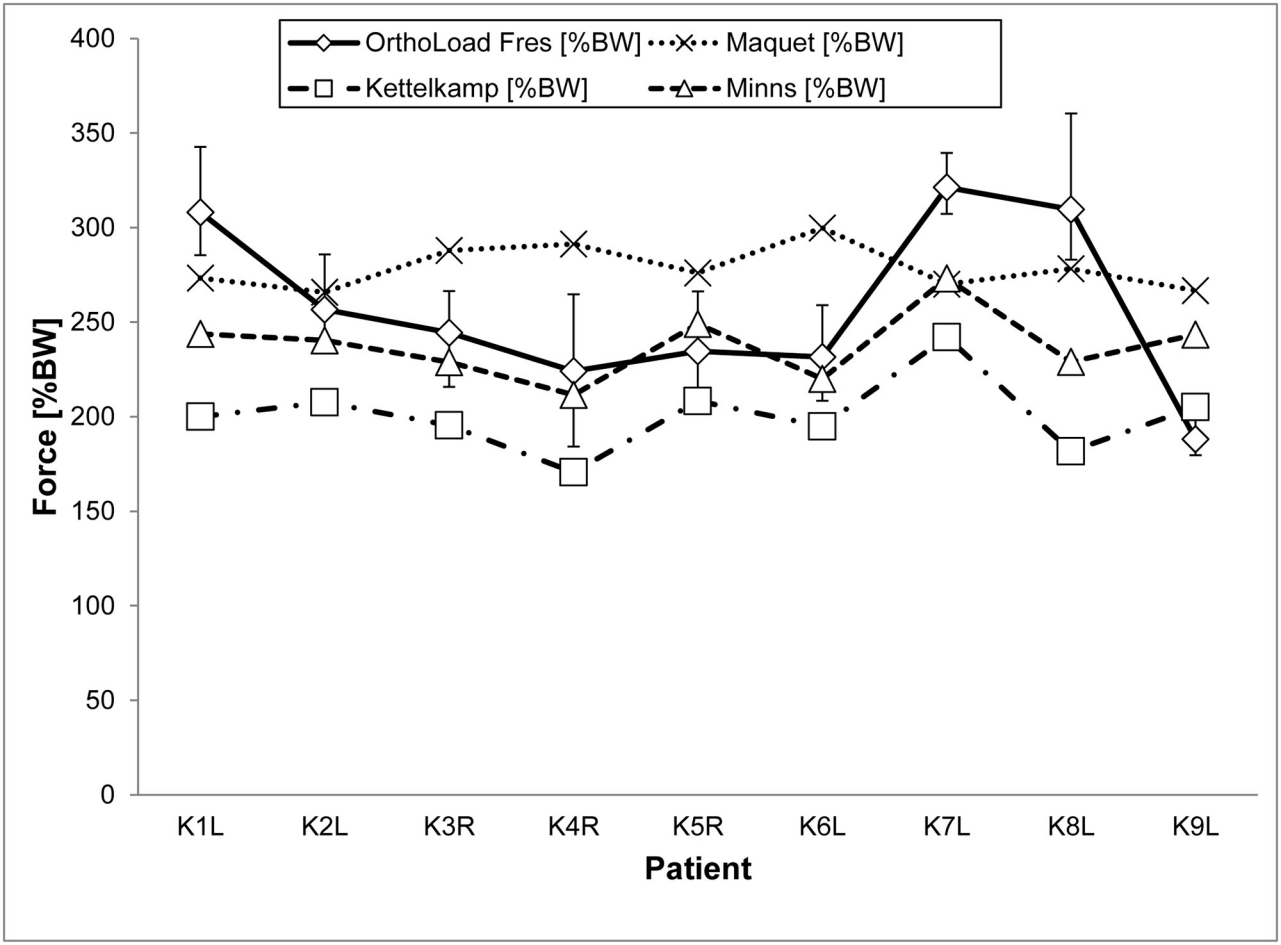
Calculated resultant knee joint forces based on the models of Maquet, Kettelkamp and Minns with corresponding *in vivo* forces for a one-leg stance. The error indicators demonstrate the minimum/maximum values. Forces in %BW.

The results of the predicted versus the *in vivo* measured joint forces for a one-leg stance are presented in Fig 7. Looking at the predictions, the values depend strongly on the different models. Kettelkamp’s model tendentially underestimates knee joint forces (approximately -17 to +128%BW). By comparison, Minns’ model is in the same range as the reference forces (-55 to 80%BW) and Maquet’s model (approximately -80 to +50%BW). The limits demonstrate a large fluctuation in the results. The RMSE was smallest for Minns’ model (43.49%BW) and highest for Kettelkamp’s model (70.15%BW) (Table 5). The absolute percentage deviations of 22.24% (Kettelkamp), 19.73% (Maquet) and 13.43% (Minns) between *in silico* and *in vivo* reflect these findings. Looking at the medial/lateral load distributions of Kettelkamp and Minns, all lateral forces are zero. Hence, a correlation analysis was omitted.

**Table 5:** Calculated resultant forces from the three models during one-leg stance including medial/lateral load distributions and *in vivo* measurements from the OrthoLoad database. Forces in %BW, RMSE in %BW and mean differences in % of the average *in vivo* forces.

Patient		K1L	K2L	K3R	K4R	K5R	K6L	K7L	K8L	K9L	
**OrthoLoad Fres [%BW]**		308.04	256.72	244.53	224.09	234.58	231.54	321.29	309.67	188.10	
**Maquet**	**FR [%BW]**	273.26	265.80	287.87	291.26	276.11	299.87	270.16	278.17	266.66	
	**Residuals [%BW]**	34.78	-9.08	-43.34	-67.17	-41.53	-68.33	51.13	31.50	-78.56	
	**RMSE [%BW]**										51.48
	**Mean [%]**										19.73
**Kettelkamp**	**F1 (lateral) [%BW]**	0.00	0.00	0.00	0.00	0.00	0.00	0.00	0.00	0.00	
	**F2 (medial) [%BW]**	200.04	207.70	195.39	170.62	208.38	194.83	242.15	181.74	205.13	
	**FR [%BW]**	200.04	207.70	195.39	170.62	208.38	194.83	242.15	181.74	205.13	
	**Residuals [%BW]**	108.00	49.02	49.14	53.47	26.20	36.71	79.14	127.93	-17.03	
	**RMSE [%BW]**										70.15
	**Mean [%]**										22.24
**Minns**	**F1 (lateral) [%BW]**	0.00	0.00	0.00	0.00	0.00	0.00	0.00	0.00	0.00	
	**F2 (medial) [%BW]**	243.80	240.37	228.82	211.53	248.98	220.00	273.19	229.00	243.47	
	**FR [%BW]**	243.80	240.37	228.82	211.53	248.98	220.00	273.19	229.00	243.47	
	**Residuals [%BW]**	64.24	16.35	15.71	12.56	-14.40	11.54	48.10	80.67	-55.37	
	**RMSE [%BW]**										43.49
	**Mean [%]**										13.43

## Discussion

The 2D biomechanical models of Maquet, Kettelkamp, and Minns able to predict knee joint forces have been individualized based on standard AP long-leg radiographs for a total of nine patients and evaluated and validated for the first time by comparing model predictions to corresponding *in vivo* force measurements.

### Model predictions

Minns’ model showed the lowest deviations to the corresponding *in vivo* measurements. The RMSEs were 21.47%BW for a static two-leg stance and 43.49%BW for a one-leg stance and the percentage deviations were 14.99% and 13.43%, respectively. In contrast, Stylianou et al. [30] reported a force difference in the superior-inferior direction of 44.9%BW during dynamic squatting compared to *in vivo* joint force measurements of a single patient. In a similar study by the same authors, the RMSE values were <29%BW for the medial/lateral contact forces over an accelerating gait cycle [31]. Marra et al. [21] found an RMSE during a gait cycle of <30%BW, Chen et al. [32] of 44.7%BW, and Lin et al. [33] of <40%BW. A low RMSE in the magnitude of 2.4%BW was achieved by Eskinazi et al. [34]. Eschweiler et al. [35] reported RMSEs from 41 to 454%BW for different 2D mathematical models of the hip for the prediction of the resultant hip joint force during a one-leg stance, considering the *in vivo* data of three patients.

Varus/valgus deformities were correctly interpreted in Minns’ model, especially in a two-leg stance, so that higher medial forces occurred in varus cases and *vice versa*. Looking at the activity intended, Kettelkamp’s model represents a two-leg stance, whereas Minns’s model was designed for a one-leg stance. Therefore, angle definitions might be inadequate and revised in the future. Comparing Kettelkamp’s predictions to the *in vivo* measurements, the model underestimates the joint forces in all situations, except for K9L in a one-leg stance. In a calculation example of Kettelkamp the resultant joint force for a 60 kg patient in one of Kettelkamp’s calculation examples was 27 kg, meaning 45%BW [24]. Although this confirms our computational results, it differs from reality [36]. Maquet’s one-leg stance model showed fluctuating results regarding the *in vivo* measurements. Thereby, a simple offset in the model output could be precluded. Although it is the simplest of the models, the calculations seem to be more reasonable than Kettelkamp’s. A major limitation of the model is that only one resultant joint force is calculated without information regarding the medial and lateral distribution, which is highly relevant for prostheses [4,5,15,16,37,38].

The correlation and regression analysis could only be reasonably performed for Kettelkamp’s and Minns‘ model during two-leg stance. Despite the high correlations observed between implant alignment and medial force ratio, the values are questionable. *In vivo* force measurements in the static condition of one-leg stance indicated a medial force ratio of 63% for neutral leg alignment [8]. The regression analysis revealed a medial force ratio of 38% for Kettelkamp’s and 31% for Minns’ model during two-leg stance.

### Clinical application

The 2D biomechanical models presented have the potential to provide patient-specific information on the knee loads (magnitude and orientation) and their distribution. This might offer the opportunity to assess the patient’s preoperative loading situation, plan and optimize implant alignment, evaluate the clinical outcome and serve as a basis for an individual rehabilitation programme in TKA. Overall optimization criteria might be the reduction of the magnitude of the resultant knee joint force and to target a predefined medial/lateral load distribution, respectively: from a technical point of view, for example, an equal distribution in order to reduce wear and provide stability. It has been shown that a carefree rectangular alignment of the implant to the mechanical leg axis might not necessarily reflect the patient’s optimum [15,16]. Due to the consideration of mechanical factors, potentially associated with prosthetic failure, the patient might benefit from higher durability and satisfaction. No additional imaging is needed for model application and the entire adaptation process is in the range of a few minutes. In comparison to *in vivo* and *in vitro* studies, they can be used to investigate the effects of changing system parameters efficiently, avoiding human or animal test subjects [22].

### Limitations

The study comprises some limitations. The models are generally restricted to two-dimensionality (coronal plane), which makes them particularly applicable for coronal alignment in preoperative TKA planning. The models assume static conditions for a one- and two-leg stance, neglecting the dynamic loads acting during activities of daily living. However, dynamic effects in slow and constant motions, such as walking, stair climbing or getting up out of a chair, might play a minor role. The muscles and soft-tissues are very simplified or neglected because of the model’s simplicity, although they have a strong impact on joint loading [39,40]. Model definitions were partly modified to allow a patient-specific adaption based on AP long-leg radiographs recorded in the standard clinical routine of TKA, which might contradict the original author’s intention. Postoperative instead of preoperative radiographs were used for model adaptation, because *in vivo* measurements are only available for TKA. This might have also accelerated and simplified the landmark acquisition process. In addition, the landmarks required were manually measured by a single observer representing a source of method error in an otherwise precise model computation [41]. Finally, *in vivo* joint forces vary between trials and subjects [42]. A one-leg stance is a demanding task, especially for elderly adults [43] and, consequently, muscle co-contraction due to instabilities might result in high knee joint forces.

## Conclusions

In conclusion, 2D biomechanical models of the knee have the potential to improve preoperative TKA planning by providing additional information of the individual loading situation to the surgeon. The patient-specific adaptation process of Maquet, Kettelkamp and Minns’ model can be performed based on AP long-leg radiographs, making them applicable in the conventional clinical workflow with acceptable costs. However, the comparison of load predictions to corresponding *in vivo* force measurements showed sobering results. Despite Minns’ model predictions being in the same magnitude as the reference, no model was able to accurately predict knee joint forces. Additionally, in one-leg stance Minns’ and Kettelkamp’s model predict unilateral medial loading, which would not occur in vivo. Hence, model optimization and more sophisticated modelling approaches are part of ongoing work, which is expected to increase the overall process time required to predict accurate knee loads. Investigations into intra- and inter-observer variability are necessary to ensure robust and clear model predictions. Finally, the clinical application requires the definition of optimization targets and meaningful clinical trials.

## Supporting information

S1 Appendix Model descriptions. (DOCX)
