# Supplementary material for: Evaluation and validation of 2D biomechanical models of the knee for radiograph-based preoperative planning in total knee arthroplasty
Source: PLoS One. 2020 Jan 8;15(1):e0227272. doi: 10.1371/journal.pone.0227272 (PMC6948753; doi:10.1371/journal.pone.0227272)
Supplement: S1 Appendix — (DOCX) [file pone.0227272.s001.docx]

# Supporting information (model descriptions)

## Maquet

Maquet’s model computes a resultant knee joint force R which depends on the force P, representing the weight and accelerations of the partial body weight and the lateral muscular force L, required for counterbalancing the one-leg stance. The centre S_7_ of the partial body weight is located on the vertical line through the centre of the ankle joint C and determines the location of P. We assessed P at:

| $P=0.929\cdot BW$ | (1) |
| --- | --- |

The muscle force L was assumed to act under an angle of $\beta=4.37^{\circ}$ relative to the tibia axis, independent of any deformities. However, we calculated an individual orientation by referring to a line connecting the most lateral point on the tibia plateau and C. The angle $\psi$ is formed by the lines of action of L and P. Thus, the static problem for the normal knee is formulated as:

| $L=P\frac{sin(\psi-\beta)}{sin(\beta)}$ | (2) |
| --- | --- |
| $R=P\frac{sin(\psi)}{sin(\beta)}$ | (3) |

Varus/valgus deformities are considered by an angle $\alpha$ of deformity between femur and tibia in the model, however, the authors did not specify any limits to differentiate between the normal and pathological situation. Therefore, we relied solely on the calculations for deformities. The calculation of R is then:

| $R=\sqrt{P^{2}+L^{2}+2PLcos(x+\beta)}$ | (4) |
| --- | --- |

Taking deformities into account, we also adjusted the calculation of the lateral muscle L:

| $L=P\frac{sin(x+\beta)}{\beta}$ | (5) |
| --- | --- |

Maquet simplified matters by the femur and tibia having approximately the same length, but in this study, the individual lengths of both have been considered. The model adaptation process is originally intended to rely on one-leg stance long-leg radiographs as input. The distance d between the centre of the hip joint H and the vertical line of action of P follows directly as model output. However, one-leg stance radiographs are rarely recorded in the standard clinical routine. Therefore, an artificial one-leg stance was approximated by predefining d. It was calculated based on the hypothesis that the centre of mass in a static one leg-stance is positioned right above the centre of the ankle joint to obtain a minimal energetic posture. We used a constant factor related to overall body height (*BH*) to approximate the individual d to:

| $d=\frac{0.191}{2}\cdot BH$ | (6) |
| --- | --- |

Despite the approximation of a one-leg stance based on two-leg stance radiographs, the two-leg stance could not be approximated without major model modifications. This is because there is only one lateral muscular force in the model to balance the partial body weight. An additional medial muscle force would need to be introduced to balance the knee in the case of valgus patients in a two-leg stance. Therefore, an implementation of a two-leg stance has been dispensed with.

## Kettelkamp

The medial and lateral knee joint forces, F_1_ and F_2_, and the tension forces, P and Q, in the idealized medial and lateral ligaments are the model output. Analogue to Maquet’s model, a distinction is made between severe and mild deformities but in a more differentiated manner due to the extensive model considering patient-specific knee joint geometry.

There is TF contact on both plateaus (two-point contact) with F_1_ and F_2_ in a normal knee. Static equilibrium is ensured by an additional vertically acting ground reaction force R and gravity force of the leg W. We approximated both forces again to:

| $R=BW$ (one-leg stance) | (7) |
| --- | --- |
| $R=0.5\cdot BW$ (two-leg stance) | (8) |
| $W=0.07\cdot BW$ | (9) |

Only one plateau surface is in contact (one-point contact) in the case of a severe varus/valgus deformity. Either the medial collateral ligament force Q or the combined lateral collateral and iliotibial tract force P is then introduced. The deformity is initially evaluated as the difference in the mechanical TF angle ($\theta_{2}-\theta_{1}$) for the force calculation. The sign of P is checked in the case of a varus tendency ($\theta_{2}-\theta_{1}<0$), expressed as follows:

| $P=\frac{W\left( X_{B}+\overline{MO}\cos\theta_{1} \right)-R\left( L_{1}\cos\theta_{1}+X_{B} \right)}{L_{1}sin\left( \theta_{2}-\theta_{1}+\Phi\right)-\overline{GS} sin\left( \theta_{2}-\theta_{1}+\Phi-\epsilon\right)+X_{B}\sin\left( \theta_{2}+\Phi\right)-Y_{B}cos(\theta_{2}+\Phi)}$ | (10) |
| --- | --- |

A severe varus deformity is assumed for a positive value of P resulting in one-point contact (F_1_ = 0). The medial force F_2_ is subsequently obtained by summing the forces in the vertical and horizontal directions:

| $F_{2}=\sqrt{F_{2x}^{2}{+F}_{2y}^{2}}$ | (11) |
| --- | --- |
| $F_{2x}=-Pcos(\theta_{2}+\Phi)$ | (12) |
| $F_{2y}=Psin\left( \theta_{2}+\Phi\right)-W+R$ | (13) |

A negative value of P indicates a mild deformity, meaning two-point contact. The joint contact force is then derived by setting P to zero and contact forces are calculated for each plateau:

| $F_{2}=\frac{W\left( \overline{MO}cos\theta_{1}+X_{A} \right)-R(L_{1}cos\theta_{1}+X_{A})}{r_{2}\sin\left( \alpha_{2}+\beta_{2} \right)-X_{A} sin( \theta_{2}-\alpha_{2})+Y_{A} \cos(\theta_{2}+\alpha_{2})}$ | (14) |
| --- | --- |

The analysis of valgus ($\theta_{2}-\theta_{1}>0$) deformities is similar. After checking the sign of Q, a one- or two-point contact is assumed with different calculations. Although the model addresses a two-leg stance, a one-leg stance was implemented. This was achieved by rotating the leg artificially. The angle of rotation was calculated based on the same assumptions used for Maquet’s model and the leg length.

## Minns

Minns investigated the effect of anatomical variations on the medial and lateral knee contact forces P_FTM_ and P_FTL_. Dimensions are taken in the sagittal plane to calculate the patellar ligament tension P_T_, which is then transferred to the frontal plane. Referring to the results of subject tests in a one-leg stance, Minns assumed P_T_ to be equal to the subject’s actual body weight:

| $P_{T}\approx BW$ | (15) |
| --- | --- |

We made additional assumptions in order to base the calculation solely on AP radiographs. The angle between the tibial axis and the vertical was estimated as 20° at 45° of knee flexion and the angle between the line of action of the patellar ligament relative to the tibia axis was idealized to 11°. It then follows that:

| $F_{V}=P_{T}\cdot cos\emptyset=BW\cdot\cos\left( 11^{\circ} \right)$ | (16) |
| --- | --- |

Since $\cos\left( 11^{\circ} \right)=0.98,$ it can be seen that the line of action of the patellar ligament relative to the tibia axis has little effect on knee contact forces, as has already been concluded by Minns. The vertical ground reaction force R was assumed, as in Kettelkamp’s model. The weight of the leg W was specified according to Minns to:

| $W=0.06\cdot BW$ | (17) |
| --- | --- |

With Minns’ model, analogue to the approach of Kettelkamp, it is possible to distinguish between severe and mild deformities. Two-point-contact with a medial and a lateral contact force is assumed in the case of less distinct deformities. Only one plateau surface is in contact in the case of severe varus/valgus deformity, which enables the calculation of one contact force. It is first necessary to determine the force in the lateral collateral ligament *P_LL_* and medial collateral ligament *P_LM_*, respectively, for severe deformities (varus/valgus). By taking the moment equilibrium about point M, the ligament force for varus angulation ($\theta_{2}-\theta_{1}<0$) is expressed through:

| $P_{LL}=\frac{W\left( \frac{T_{M}}{2}+\frac{L_{1}}{2}\cos\theta_{1} \right)\cos\psi-R\left( L_{1}\cos\theta_{1}+\frac{T_{M}}{2} \right)\cos\psi-F_{V}\sin\theta_{2}\frac{T_{M}}{2}}{\left( \frac{T_{M}}{2}+\frac{T}{2} \right)\cos\beta_{L}}$ | (18) |
| --- | --- |

A positive value implies a tension force in the lateral collateral ligament, resulting in one-point contact. By summing the forces acting vertically the medial contact force is calculated as follows:

| $P_{FTM}=\frac{P_{LL}\cos\beta_{L}+F_{V}\sin\theta_{2}+(R-W)\cos\psi}{\cos\theta_{M}}$ | (19) |
| --- | --- |

The procedure for valgus angulation ($\theta_{2}-\theta_{1}>0$) is analogous. The resulting one- or two-point contact can be determined by checking the sign of the force in the medial collateral ligament *P_LM_*, followed by different calculations. A negative value of *P_LL_* and *P_LM_*, respectively, indicates a less distinct deformity with two-point TF contact. The force in the corresponding collateral ligament is set to be zero and both contact forces on each plateau are calculated by taking moments about the knee joint centre (O):

| $P_{FTM}=\frac{\left( F_{V}\sin\theta_{2}+(R-W)\cos\psi\right)\frac{T_{L}}{2}-R L_{1}\cos\theta_{1}\cos\psi+W\frac{L_{1}}{2}\cos\theta_{1}\cos\psi}{\left( \frac{T_{L}}{2}+\frac{T_{M}}{2} \right)\cos\theta_{M}}$ | (20) |
| --- | --- |
| $P_{FTL}=\frac{\left( F_{V}\sin\theta_{2}+(R-W)\cos\psi\right)\frac{T_{M}}{2}+R L_{1}\cos\theta_{1}\cos\psi-W\frac{L_{1}}{2}\cos\theta_{1}\cos\psi}{\left( \frac{T_{L}}{2}+\frac{T_{M}}{2} \right)\cos\theta_{M}}$ | (21) |

A one-leg stance was approximated in the same way as that described for Kettelkamp’s model.
